# Additivity of the Stabilization Effect of Single Amino Acid Substitutions in Triple Mutants of Recombinant Formate Dehydrogenase from the Soybean Glycine max

**Published:** 2015

**Authors:** A. A. Alekseeva, I. S. Kargov, S. Yu. Kleimenov, S. S. Savin, V. I. Tishkov

**Affiliations:** A.N.Bach Institute of Biochemistry, Federal Research Center “Fundamentals of Biotechnology” of the Russian Academy of Sciences, Leninskiy Prospect, 33/2, Moscow,119071, Russia; Innovations and High Technologies MSU Ltd, Tsimlyanskaya St., 16, Moscow, 109559, Russia; Departament of Chemistry, M.V. Lomonosov Moscow State University, Leninskie Gory, 1/3, Moscow, 119991, Russia; N.K. Koltsov Institute of Developmental Biology of the Russian Academy of Sciences, Vavilova St., 26, Moscow, 119334, Russia

**Keywords:** protein engineering, multi-point mutants, rational design, stabilization, stability, synergistic effect, formate dehydrogenase, Glycine max

## Abstract

Recently, we demonstrated that the amino acid substitutions Ala267Met and Ala267Met/Ile272Val (Alekseeva *et al*., Biochemistry, 2012), Phe290Asp, Phe290Asn and Phe290Ser (Alekseeva *et al*., Prot. Eng. Des. Select, 2012) in recombinant formate dehydrogenase from soya *Glycine max *(SoyFDH) lead to a significant (up to 30–100 times) increase in the thermal stability of the enzyme. The substitutions Phe290Asp, Phe290Asn and Phe290Ser were introduced into double mutant SoyFDH Ala267Met/Ile272Val by site-directed mutagenesis. Combinations of three substitutions did not lead to a noticeable change in the catalytic properties of the mutant enzymes. The stability of the resultant triple mutants was studied through thermal inactivation kinetics and differential scanning calorimetry. The thermal stability of the new mutant SoyFDHs was shown to be much higher than that of their precursors. The stability of the best mutant SoyFDH Ala267Met/Ile272Val/Phe290Asp turned out to be comparable to that of the most stable wild-type formate dehydrogenases from other sources. The results obtained with both methods indicate a great synergistic contribution of individual amino acid substitutions to the common stabilization effect.

## INTRODUCTION

NAD(P)^+^-dependent formate dehydrogenases ([1.2.1.2], FDHs) have been found in bacteria, yeast, fungi, and plants [1-3]; however, plant FDHs have been far less studied than enzymes from microorganisms. Our laboratory has actively studied plant recombinant formate dehydrogenases, including FDH from the soybean* Glycine max *(SoyFDH) [3-7]. A plasmid vector was constructed that enabled expression of the soluble and active SoyFDH in *Escherichia coli *cells [8]. Increased interest in this enzyme stems from the fact that the Michaelis constant values of SoyFDH both for NAD^+^ and formate are lower than those of formate dehydrogenases from other sources (*Table 1*). Systematic studies of various formate dehydrogenases [2] and analysis of the structure-function relationship have revealed a number of amino acid residues that affect the stability and catalytic properties of soybean formate dehydrogenase [3-7]. More than 20 mutant SoyFDHs were prepared using site-directed mutagenesis. More than half of them had a higher thermal stability compared to the one for the wild-type enzyme, while the Michaelis constants practically didn’t change. The most interesting results were obtained using enzyme stabilization approaches such as filling the cavity inside the protein globule [4] and substitution of a hydrophobic residue by hydrophilic ones on the protein globule surface [5, 6]. SoyFDHs with a substitution of one or two amino acid residues, Ala267Met and Ala267Met/Ile272Val, respectively, were produced using the first approach. In this case, the thermal stability of a double mutant was significantly higher compared to that of its precursor [4]. In the case of the second approach, a hydrophobic residue, Phe290, located in the coenzyme-binding domain on the surface of the protein globule was replaced by eight other amino acid residues [5, 6]. In this work, three triple mutants were produced by introducing the point substitutions Phe290Asp, Phe290Asn, and Phe290Ser into double mutant Ala267Met/Ile272ValSoyFDH. The Phe290Asp substitution providing the strongest stabilization effect was assumed to produce a triple mutant with the highest stability. The other two triple mutants were obtained to elucidate how the differences in the stabilization effect at the 290th position will affect the overall stability and catalytic properties of SoyFDH with three amino acid substitutions.

**Table 1:** Kinetic parameters of wild-type and mutant SoyFDHs compared to those of formate dehydrogenases from other sources

Enzyme	k_cat_, s^–1^	K_m_^formate^, mM	K_m_^NAD+^, μM	k_cat_/K_m_^NAD+^, (μM*s)^–1^	k_cat_/K_m_^formate^, (μM*s)^–1^	Reference
wt-SoyFDH	2.9	1.5	13.3	0.22	1.93	[4-6]
SoyFDHM1 (A267M)	5.0	2.1	9.9	0.51	2.38	[4]
SoyFDH M1+M2 (A267M/I272V)	2.2	2.4	13.3	0.17	0.92	[5]
SoyFDH M3 (F290N)	2.8	4.5	14.0	0.40	1.02	[5]
SoyFDH M4 (F290D)	5.1	5.0	12.8	0.20	0.62	[5]
SoyFDH M5 (F290S)	4.1	4.1	9.1	0.45	1.00	[5]
SoyFDH M1+M2+M3 (A267M/I272V/F290N)	3.2±0.2	2.2±0.3	14.1±0.7	0.23	1.45	Present study
SoyFDH M1+M2+M4 (A267M/I272V/F290D)	2.9±0.2	2.8±0.4	20.3±1.3	0.14	1.04	Present study
SoyFDH M1+M2+M5 (A267M/I272V/F290S)	3.7±0.1	2.3±0.3	16.1±0.4	0.23	1.61	Present study
wt-AthFDH	3.8	2.8	50	0.08	1.36	[8]
wt-LjaFDH	1.2	6.1	25.9	0.05	0.20	[9]
wt-CboFDH	3.7	5.9	45	0.08	0.63	[10,11]
wt-MorFDH	7.3	7.5	80	0.09	0.97	[2]
wt-PseFDH	7.3	6.5	65	0.11	1.12	[2]
PseFDHGAV	7.3	6	35	0.21	1.22	[2,3]
PseFDHSM4	7.3	3.2	41	0.18	2.28	Own data

PseFDH, MorFDH, CboFDH, AraFDH, and LjaFDH are formate dehydrogenases from bacteria Pseudomonas sp.101 and Moraxella sp. C1, yeast Candida boidinii, and plants Arabidopsis thaliana and Lotus japonicus, respectively.

## MATERIALS AND METHODS

Molecular-biology-grade reagents were used for genetic engineering experiments. Bactotryptone, yeast extract, and agar (Difco, USA), glycerol (99.9%) and calcium chloride (ultra pure), dipotassium hydrogen phosphate, sodium dihydrogen phosphate (pure for analysis), lysozyme (Fluka/BioChemika, Switzerland), lactose (analytical grade), ampicillin and chloramphenicol (Sigma, USA), and glucose and sodium chloride (analytical grade (Helicon, Russia) were used in microbiological experiments. Restriction endonucleases, phage T4 DNA ligase, and Pfu DNA polymerase (Fermentas, Lithuania) were used for cloning DNA fragments and site-directed mutagenesis. Reagent kits (Fermentas, Lithuania) were used to isolate DNA from agarose gel and plasmids from *E. coli *cells. Oligonucleotides for PCR and sequencing were synthesized by the Syntol company (Russia). Water purified by a MilliQ device (Millipore, USA) was used in these experiments.

All reagents used in protein electrophoresis were manufactured by Bio-Rad (USA). NAD^+^ with a purity ≥ 98% (AppliChem, Germany), sodium formate and EDTA (Merck, Germany), sodium azide (Sigma, Germany), ammonium sulfate (chemically pure grade, DiaM, Russia), sodium dihydrogen phosphate, disodium phosphate, and urea (analytical grade, Reakhim, Russia) were used to isolate an enzyme and examine its properties.

**Site-directed mutagenesis reaction**

Site-directed mutagenesis enabling Phe290Asp, Phe290Asn, and Phe290Ser substitutions in the Soy- FDH amino acid sequence were introduced as previously described [5]; however, instead of the pSoyFDH2 plasmid with the wild-type SoyFDH gene, the pSoyFDH2_M1M2 plasmid was used as an initial template. This plasmid contains a gene encoding SoyFDH with Ala267Met and Ile272Val substitutions.

For each mutant plasmids were isolated from three colonies. The correctness of mutation introduction was proved by sequencing of the plasmid DNA at the Genome Center for Collective Use (Engelhardt Institute of Molecular Biology, Moscow).

**Expression of mutant SoyFDHs in *E. coli *cells**

Wild-type SoyFDH and its mutant forms were expressed in *E.coli *BL21(DE3) Codon Plus/pLysS cells. To generate a producer strain, the cells were transformed with an appropriate plasmid and plated on Petri dishes with an agar medium containing ampicillin (150 μg/mL) and chloramphenicol (25 μg/mL). To prepare the inoculum, a single colony was taken from a plate and cultured at 30 °C overnight in 4 mL of a modified 2YT medium (10 g/L yeast extract, 16 g/L bactotryptone, 1.5 g/L sodium dihydrogen phosphate, 1 g/L dipotassium hydrogen phosphate, pH 7.5) in the presence of 150 μg/mL ampicillin and 25 μg/mL chloramphenicol. Then, the cells were subcultured into a fresh medium (1 : 100 dilution) and cultured at 37 °C to A_600_≈ 0.6–0.8. The inoculum (10% of the total medium volume) was added into conical 1L baffled flasks. The cells were cultured at 30 °C and 80–90 rpm until an absorbance of A_600_=0.6–0.8. Then, the cells were induced by adding a solution of lactose (300 g/L) to a final concentration of 20 g/L. After induction, the cells were cultured for 17 h and then pelleted using an Eppendorf 5403 centrifuge (20 min, 5,000 rpm, 4 °C). The resulting biomass was re-suspended in a 0.1 M sodium phosphate buffer pH 8.0 at a 1 : 4 (weight-volume) ratio. The resulting suspension was frozen and stored at –20 °C.

**Isolation and purification of mutant enzymes**

To isolate mutant SoyFDHs, a 20% cell suspension in a 0.1 M sodium phosphate buffer, pH 8.0, was subjected to two cycles of freezing-thawing; then, the cells were disrupted using a Branson Sonifier 250 ultrasonic cell disruptor (Germany) under continuous cooling. The precipitate was removed by centrifugation on an Eppendorf 5804R centrifuge (11,000 rpm, 30 min).

The enzyme purification procedure included precipitation of ballast proteins with ammonium sulfate (saturation of 40%), precipitation of the target protein (with ammonium sulfate saturation of 85%) and its subsequent reconstitution in a solution containing 45% ammonium sulfate, hydrophobic chromatography on Phenyl Sepharose, and desalting on a Sephadex G-25 column [4, 5]. The sample purity was monitored by analytical electrophoresis in a 12% polyacrylamide gel in the presence of 0.1% sodium dodecyl sulfate (Bio-Rad electrophoresis apparatus).

**Formate dehydrogenase activity measurement**

The enzymatic activity was determined spectrophotometrically by the absorbance of NADH at 340 nm (ε_340_=6,220 M^–1^cm^–1^) on a Schimadzu UV1800PC spectrophotometer at 30 °C in 0.1 M sodium phosphate buffer, pH 7.0, containing 0.3 M sodium formate and 0.4 mg/mL NAD^+^.

**Determination of the Michaelis constant**

Michaelis constants for NAD^+^ and formate were determined spectrophotometrically by measuring the dependence of enzymatic activity on the concentration of one of the substrates in a range from 0.3 up to 6–7 K_M_ at saturating concentrations of the second substrate ( > 20 K_M_). The exact concentration of initial NAD^+^ solutions was measured at 260 nm (ε_260_ = 17,800 M^–1^cm^–1^). The exact concentration of sodium formate was determined enzymatically using formate dehydrogenase, based on the formation of NADH caused by oxidation of the formate ion to CO_2_. 50 μL of a NAD^+^ solution (20 mg/mL in 0.1 M phosphate buffer, pH 8.0), 20 μL of a formate dehydrogenase solution (50 U/mL), and 0.1 M phosphate buffer, pH 8.0, were added to a quartz spectrophotometric cuvette (total and reaction volumes were 4 and 2 mL, respectively) to a total volume of 1.96 mL. The cell was incubated at 37 °C for 15 min, and then the absorbance at 340 nm was determined. 0.1 mL of a 3 M sodium formate solution in 0.1 M phosphate buffer, pH 7.0, prepared by weight in a volumetric flask was added to a 100 mL volumetric flask with 0.1 M phosphate buffer, pH 8.0, using a 0.1 mL glass pipette and adjusted to a volume of 100 mL with the same buffer. The resulting solution was stirred, and a 40 μL sample of the solution was added to the cell with the reaction mixture. Upon completion of the reaction (15–20 min), the solution absorbance was measured. The absorbance was subtracted with an initial absorbance value, and the resultant difference was used to calculate the exact concentration of sodium formate. *K*_M_ values were determined by nonlinear regression using the Origin Pro 8.5 software.

**Determination of catalytic constants**

The catalytic constant values were calculated from the dependence of the activity of several enzyme samples on the active site concentrations of the samples by linear regression using the Origin Pro 8.5 software. The concentrations of active sites were determined by measuring quenching of enzyme fluorescence by NAD^+^ and the azide ion [7]. Measurements were performed in 0.1 M sodium phosphate buffer, pH 7.0, on a Cary Eclipse fluorimeter (Varian, USA).

**Thermal stability analysis based on the thermal inactivation kinetics**

The enzyme thermal stability was studied in 0.1 M sodium phosphate buffer, pH 7.0, containing 0.01 M EDTA. Series of 1.5 mL plastic test tubes with 50 μL of an enzyme solution (0.2 mg/mL) in each were prepared for each experiment. The tubes were placed in a water thermostat pre-heated to a required temperature (46–66 °C, temperature accuracy of ± 0.1 °C). At a certain time point, one tube was taken out and transferred into ice for 5 min, after which the tube was centrifuged at 12,000 rpm on an Eppendorf 5415D centrifuge for 3 min. The residual formate dehydrogenase activity was measured as described above. The thermal inactivation rate constant *k_in_*was determined as the slope of the natural logarithm of residual activity vs time (semilogarithmic coordinates, ln(*A*/*A_0_*) –*t*)) by linear regression using the Origin Pro 8.5 software.

**Analysis of the enzyme thermal stability using differential scanning calorimetry**

Differential scanning calorimetry experiments were performed using a DASM-4 adiabatic differential scanning microcalorimeter (Biopribor, Bach Institute of Biochemistry, Federal Research Center “Fundamentals of Biotechnology”, Russia). The reaction volume of platinum capillary calorimetric cells was 0.48 mL. To prevent the formation of air bubbles and evaporation of solutions at elevated temperatures, 2.2 atm extra pressure was maintained in the calorimetric cells. The instrument calibration was carried out by feeding one cell to a fixed power (Δ*W *= 25 μW).

Before a calorimetric experiment, the temperature drift of instrument readings was determined. During measurement, the blank cell contained 0.1 M sodium phosphate buffer, pH 7.0, and the sample cell contained SoyFDH dissolved in the same buffer. The enzyme concentration was 2.0 mg/mL, and the heating rate was 1 °C/min.

The thermal denaturation reversibility was analyzed by re-scanning of a sample after its cooling to 10–12 °C directly in a calorimeter. The absence of a denaturation peak upon repeated measurements confirmed irreversible denaturation.

Processing and analysis of denaturation curves were performed according to a standard procedure by means of special macroses using the Matlab 8.0 software. The calorimeter drift and a step change in the heat capacity associated with the denaturation completeness were subtracted from the measured data before calculating the denaturation parameters. The calorimetric specific heat capacity (*ΔC_p_*) was calculated based on the area under the curve of the protein excess heat capacity vs temperature; the denaturation (melting) temperature* T*_m_ was determined as the temperature at the maximum on the same curve. The error of *ΔC_p_*calculation was 5–8%. The experimental error of the *T*_m_ measurement did not exceed 0.2 °C.

**Computer simulation**

The SoyFDH structure was analyzed using the Accelrys Discovery Studio 2.1 software package. The same package was used to prepare images of the protein globule.

## RESULTS AND DISCUSSION

**Fig. 1:**
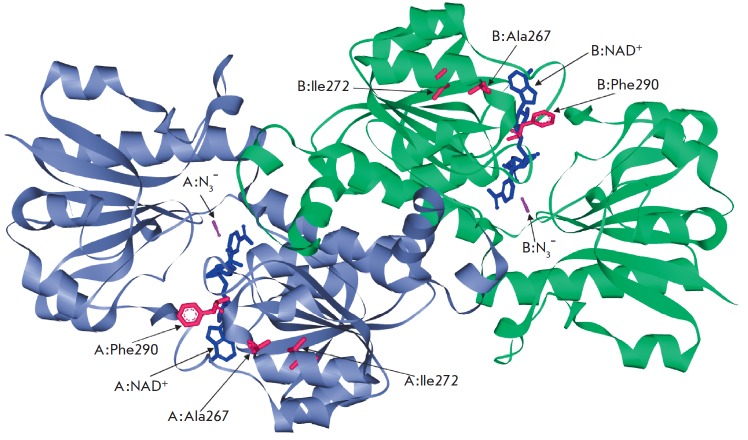
3D-structure of a ternary complex of SoyFDH with coenzyme NAD^+^ and strong inhibitor N_3_^-^

As already noted, triple mutant SoyFDHs were produced by introduction of three amino acid residues (Asp, Asn, and Ser) into the 290th position of a double mutant with Ala267Met/Ile272Val substitutions. *Fig. 1* presents the structure of the [SoyFDH-NAD^+^-N3^–^] ternary complex indicating the positions of the Ala267, Ile272, and Phe290 residues selected for site-directed mutagenesis in the protein globule. As seen from the *Fig. 1*, all three residues are located in the coenzyme-binding domain; however, the first two residues are more distant from the NAD^+^ molecule than the Phe290 residue. The substitutions at the 290th position had a much more noticeable effect on both the catalytic properties and the thermal stability compared to the replacements at positions 267 and 272 [4-6]. We assumed that a combination of the three amino acid substitutions would provide a more stable mutant SoyFDH. For convenience purposes, the Ala267Met, Ile272Val, Phe290Asn, Phe290Asp, and Phe290Ser substitutions are thereafter designated as M1, M2, M3, M4, and M5, respectively.

**Production of mutant SoyFDHs**

Substitutions of the nucleotides responsible for the desired mutations were performed by a polymerase chain reaction. Three plasmids for each of the three mutants were isolated. According to sequencing, the* soyfdh *gene in all plasmids contained the desired mutations only. Plasmids encoding the *soyfdh *gene with mutations leading to amino acid substitutions (A267M/ I272V/F290N), (A267M/I272V/F290D), and (A267M/ I272V/F290S) were used to transform the *E. coli* BL21(DE3)CodonPlus/pLysS strain. All three mutant SoyFDHs were demonstrated to be expressed in the active and soluble forms in recombinant strains. According to analytical polyacrylamide gel electrophoresis in the presence of sodium dodecyl sulfate, the purity of the isolated SoyFDH samples was not less than 95%.

**The kinetic properties of the mutant enzymes**

*Table 1* shows the values of the catalytic constant and Michaelis constants for NAD^+^ and formate for the three new multi-point mutant SoyFDHs, as well as similar values for mutant precursors and some other bacterial, yeast, and plant formate dehydrogenases. As seen from *Table 1*, the introduction of an additional substitution into the 290^th^ position of a double mutant has no effect on the Michaelis constant for formate, whereas the *K*_M_ value for NAD^+^ is either comparable to or higher than that of a double mutant precursor. These data are well correlated with the fact that all mutable residues are located in the coenzyme-binding domain. The catalytic constant of a double mutant is less than that of point mutants with substitution at the 290^th^ position. Combination of the three amino acid replacements leads to the *k_cat_*value of triple mutants being either comparable or higher than that of the double mutant but less than that of point mutants with the substitutions Phe290Asp and Phe290Asn. Also, the lack of correlation between the catalytic properties of double, triple, and point mutants should be noted. For example, mutant SoyFDH Phe290Asp has the highest *k_cat_*value among point mutants with a substitution at the 290^th^ position, while the triple mutant containing this substitution has the lowest catalytic constant among multi-point mutants.

In summary, it can be concluded that the kinetic parameters and catalytic properties of SoyFDHs with the substitutions Ala267Met/Ile272Val/Phe290Asn and Ala267Met/Ile272Val/Phe290Ser remained at the level of the wild-type enzyme and mutant formate dehydrogenases from *Pseudomonas *sp. 101, PseFDH GAV and PseFDH SM4 (*Table 1*); in mutant SoyFDH A267M/I272V/F290D, these parameters slightly deteriorated but still remained better than in CboFDH, which is widely used at present.

**Analysis of the thermal stability of mutant SoyFDHs through thermal inactivation kinetics**

**Fig. 2:**
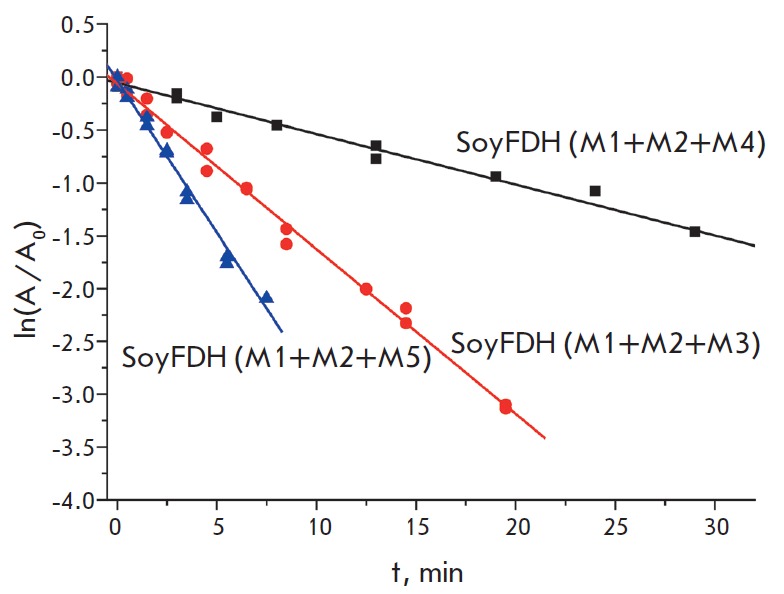
Dependence of the natural logarithm of the residual activity on time for triple mutant SoyFDHs at 64 °C, 0.1 M sodium phosphate buffer, pH 7.0. M1 – Ala267Met, M2 – Ile272Val, M3 – Phe290Asn, M4 – Phe290Asp, M5 – Phe290Ser

**Fig. 3:**
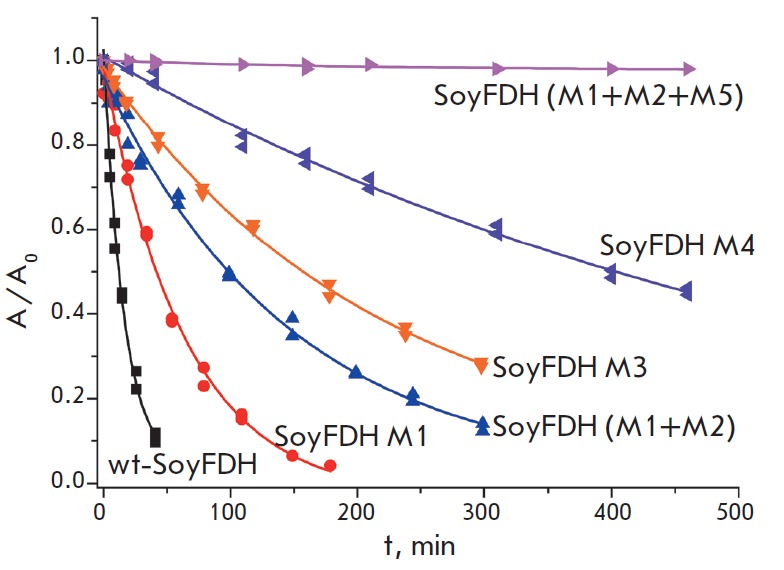
Dependence of the residual activity on time for wild-type (wt-SoyFDH) and several mutant SoyFDHs at 54 °C. 0.1 M sodium phosphate buffer, pH 7.0. M1 – Ala267Met, M2 – Ile272Val, M3 – Phe290Asn, M4 – Phe290Asp, M5 – Phe290Ser

The thermal inactivation kinetics of mutant SoyFDHs with the substitutions Ala267Met/Ile272Val/ Phe290Asn and Ala267Met/Ile272Val/Phe290Ser was studied in a temperature range of 58–64 °C, while Soy- FDH Ala267Met/Ile272Val/Phe290Asp was analyzed in a range of 60–66 °C. Selection of the temperature range was based on the higher stability of the last mutant; therefore, higher temperatures had to be used for achieving the time intervals required for a decrease in activity similar to that observed in other mutants. The inactivation kinetics in the entire temperature range followed the first-order kinetics. The thermal inactivation rate constants were calculated from the slopes of these straight lines. The thermal inactivation rate constant value did not depend on the enzyme concentration in the entire temperature range, indicating the true monomolecular mechanism of the thermal inactivation process. *Fig. 2* presents the dependence of the natural logarithm of the residual activity of the three new mutant enzymes on time at 64 °C. It is seen that mutant SoyFDH with the Ala267Met/Ile272Val/Phe290Asp substitutions has a higher stability than the other two triple mutants. Unfortunately, the inactivation curves of the precursor mutants could not be obtained at this temperature, since they were almost completely inactivated in less than 5 min under these conditions. To illustrate the stabilization effect, *Fig. 3* shows the dependencies of the residual activity of several mutant SoyFDHs on time at 54 °C. It is seen that Ala267Met/ Ile272Val/Phe290Asp SoyFDH is almost inactivated for about 8 h, while the half-inactivation period of the wild-type enzyme and mutant SoyFDHs with the Ala267Met, Ala267Met/Ile272Val, and Phe290Asp substitutions is 19, 56, 153, and 460 min, respectively. Thus, we can draw a conclusion about the large additive effect of the substitution introduced into the 290^th^ position in Ala267Met/Ile272Val double mutant SoyFDH on an increased enzyme thermal stability.

**Fig. 4:**
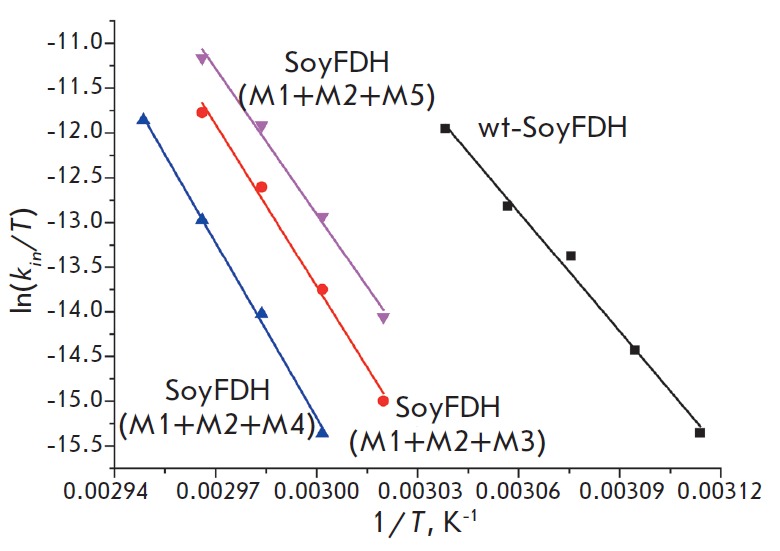
Dependence of the inactivation rate constants on temperature in coordinates ln(*k_in_*/*T*) – 1/*T *for wild-type and mutant SoyFDHs. 0.1 M sodium phosphate buffer, pH 7.0. M1 – Ala267Met, M2 – Ile272Val, M3 – Phe290Asn, M4 – Phe290Asp, M5 – Phe290Ser

*Fig. 4* shows the temperature dependence of the thermal inactivation rate constants for mutant SoyFDHs with triple substitutions and the wild-type enzyme in the coordinates ln(*kin/T*) vs *1/T*, where *T *is the temperature in Kelvin. These coordinates are a linear anamorphosis for the equation of the temperature dependence of the rate constant for the transition state theory [12]:

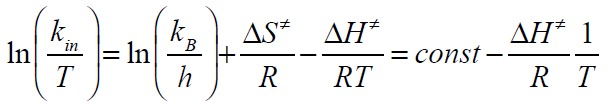



where *k_B_*and *h *are the Planck and Boltzmann constants, respectively; *R *is the universal gas constant; and Δ*H*^≠^ and Δ*S*^≠^ are activation parameters.

The linear form of the resulting dependences suggests that the dependence of the thermal inactivation rate constants of native SoyFDH and the mutant forms is actually described by the transition state theory equation.

*Table 2* provides the values of the Δ*H*^≠^ and Δ*S*^≠^ activation parameters for the thermal inactivation process that are derived from the temperature dependences of the thermal inactivation rate constants using the equation from the transition state theory. It can be seen that the activation enthalpy Δ*H*^≠^ and entropy Δ*S*^≠^ values for the enzyme with an Ala267Met/Ile272Val/Phe290Asp triple substitution are the highest ones among all tested FDH mutants and are almost the same as those of one of the most thermostable FDH from *Pseudomonas *sp. 101 (PseFDH). It should be noted that the Δ*H*^≠^ and Δ*S*^≠^ values of the mutant with the Ala267Met/Ile272Val/ Phe290Asp substitutions are higher than those of formate dehydrogenase from yeast *Candida boidinii *and plant *Arabidopsis thaliana*.

**Table 2:** Activation parameters ΔH^≠^ and ΔS^≠^ for thermal inactivation of wild-type and mutant SoyFDHs and wild-type formate dehydrogenases from various sources (0.1 M sodium phosphate buffer, pH7.0)

Enzyme	ΔH^≠^, kJ/M	ΔS^≠^, J/(M*K)	Reference
wt-SoyFDH	370 ± 20	830 ± 60	[4]
SoyFDHM1(A267M)	400	900	[4]
SoyFDH M1+M2 (A267M/I272V)	450±30	1,040±80	[4]
SoyFDHM5 (F290D)	520±30	1,240±70	[5]
SoyFDHM3 (F290N)	450±20	1,050±60	[5]
SoyFDHM5 (F290S)	440±20	1,020±70	[5]
SoyFDH M1+M2+M3 (A267M/I272V/F290N)	500±30	1,190±90	Present study
SoyFDH M1+M2+M4 (A267M/I272V/F290D)	540±20	1,310±50	Present study
SoyFDH M1+M2+M5 (A267M/I272V/F290S)	450±30	1,050±80	Present study
wt-AthFDH*	490	1,200	[2]
wt-PseFDH*	570	1,390	[2]
wt-CboFDH*	500	1360	[13]
wt-SceFDH*	420	n.d.**	[14]

* AthFDH, PseFDH, CboFDH, and SceFDH are formate dehydrogenases from plant A. thaliana, bacterium Pseudomonassp.101,and yeast C. boidinii and Saccharomices cerevisiae, respectively.

**n.d. – no data

As seen from *Table 2*, the activation enthalpy of wild-type SoyFDH is lower than that of its mutants. This means that the thermal inactivation rate constant of all mutant SoyFDHs will decrease faster than that of wild-type SoyFDH as the temperature decreases: i.e. the stabilization effect should increase with decreasing temperature. The thermal inactivation rate constant and stabilization effect values were calculated in a wide temperature range using the transition statetheory equation and Δ*H*^≠^ and Δ*S*^≠^ values obtained for wild-type SoyFDH and mutant enzymes. *Table 3*shows the stabilization effect values of mutant SoyFDHs compared to the wild-type enzyme. It is seen that the stabilization effect in the most stable mutant enzyme with the Ala267Met/Ile272Val/Phe290Asp substitutions ranges from 2,330 to 51 times at elevated temperatures (46–66 °C). It is much more than in the most successful point mutant Phe290Asp. Therefore, the Ala267Met/ Ile272Val/Phe290Asp mutant is the most thermostable mutant among the mutant SoyFDHs described in this paper. Its thermal stability is higher than that of the formate dehydrogenases from *A. thaliana *and *C. boidinii*.

Since all the mutable residues are located in the coenzyme- binding domain, it was interesting to estimate the contribution of substitution at the 290^th^ position to the overall stabilization effect of a triple mutant. The additivity concept is used to assess such a contribution. For this purpose, an experimental value of the stabilization effect is compared to a theoretically possible value. If the stabilization effects in the original mutants are independent of each other, the theoretical effect of the stabilization of a mutant that combines the analyzed substitutions will be equal to the product of the stabilization effects of the original mutants. Coincidence of the theoretical and experimental values means 100% additivity. If this value is less than 100%, then the additivity is not complete, and if it is more than 100%, then there is positive cooperativity or synergism of the stabilization effect. Columns 7, 9, and 11 of *Table 3* show values of the theoretical stabilization effect calculated as a multiplication of the stabilization effect value of the initial mutant SoyFDH with an Ala267Met/Ile272Val double substitution on the stabilization effect value of a mutant with an appropriate substitution at the 290^th^ position. As seen from columns 6 and 7, in the case of mutant SoyFDH Ala267Met/Ile272Val/Phe290Asn, a 100% additivity is observed at 64 °C, while lowering the temperature leads to a slow decrease in this parameter. A similar pattern is observed for mutant SoyFDH Ala267Met/Ile272Val/ Phe290Ser (*Table 3*; columns 11 and 12) at 62 °C and below; while at 64 °C, the stabilization additivity is greater than 100%. A very interesting situation is observed for the most stable mutant SoyFDH Ala267Met/Ile272Val/ Phe290Asp (*Table 3*; columns 9 and 10). The stabilization additivity exceeds 100% at all tested temperatures; however, this parameter is reduced with a decrease in temperature, like in the two previous cases. High additivity of the stabilization effect (up to 100%) upon combination of several amino acid substitutions was also observed for FDH from bacterium *Pseudomonas *sp. 101 a [15, 16], but the magnitudes of the stabilization effects are not comparable (1.1–2.5 times) with the effects observed in this work.

**Table 3:** Stabilization effect* for mutant SoyFDHs compared to the wild-type enzyme at various temperatures (0.1 M sodium phosphate buffer, pН7.0)

T, °C	Stabilization effect											
	1	2	3	4	5	6	7**	8	9**	10	11**	12
	wt-SoyFDH	SoyFDH М1 (A267M) [1]	SoyFDH М1+М2 (A267M/I272V) [1]	SoyFDH М3 (F290N) [2]	SoyFDH М4 (F290D) [2]	SoyFDH М5 (F290S) [2]	Stab.eff. (М1+М2)*Stab. eff. М3	SoyFDH М1+М2+М3	Stab.eff. (М1+М2)*Stab. eff. М4	SoyFDH М1+М2+М4	Stab.eff. (М1+М2)*Stab. eff. М5	SoyFDH М1+М2+М5
25	1	9	130	200	11,000	52	26,000	9.200	1,430,000	233,200	6,760	450
30	1	7	81	120	4,100	33	9,720	3,820	332,100	73,540	2,673	265
46	1	4.3	18	25	230	8.8	450	280	4,140	2,330	158	54
48	1	4	15	20	160	7.5	300	188	2,400	1,440	114	41
50	1	3.8	13	17	120	6.5	221	148	1,560	1,030	85	37
52	1	3.6	11	15	85	5.5	165	135	935	856	61	38
54	1	3.4	8.9	12	61	4.8	107	76	5,439	436	43	24
56	1	3.2	7.5	10	44	4.1	75	58	330	308	30.8	21
58	1	3	6.4	8.6	32	3.5	55	50	205	218	22	19
60	1	2.8	5.4	7.3	23	3.1	39	32	124	160	17	14
52	1	2.7	4.6	6.1	17	2.7	28	23	78	93	12	11
64	1	2.5	3.9	5.2	12	2.3	20	22	47	71	9	12
66	1							15		51		9

*Stabilization effect was calculated as the (k_in_)^wt^/(k_in_)^mut^ ratio at the same temperature. Values shown in bold were calculated using experimental constants. The other values of the stabilization effect were calculated using the transition state theory equation and appropriate activation parameters ΔH^≠^ and ΔS^≠^ from Table 2.

**Columns 7, 9 and 11 show the theoretical stabilization effect in the case of 100% additivity. These values were calculated as multiplication of the stabilization effect for double mutant Soy FDH (М1+М2) and the stabilization effect for a mutation at the 290th position.

The cause for increasing theoretical stabilization effect (and, consequently, reducing effect of additivity) with lowering temperature is not yet clear, but it should be noted that the thermal inactivation rate constant of various SoyFDH mutants is differently dependent on the temperature, and a total change in the protein structure caused by the combination of amino acid substitutions may be different at different temperatures. Further experiments, which were beyond the goals and objectives of our work, will provide a more precise understanding of the causes of the observed effect.

**Analysis of the thermal stability of mutant SoyFDHs by differential scanning calorimetry**

**Fig. 5:**
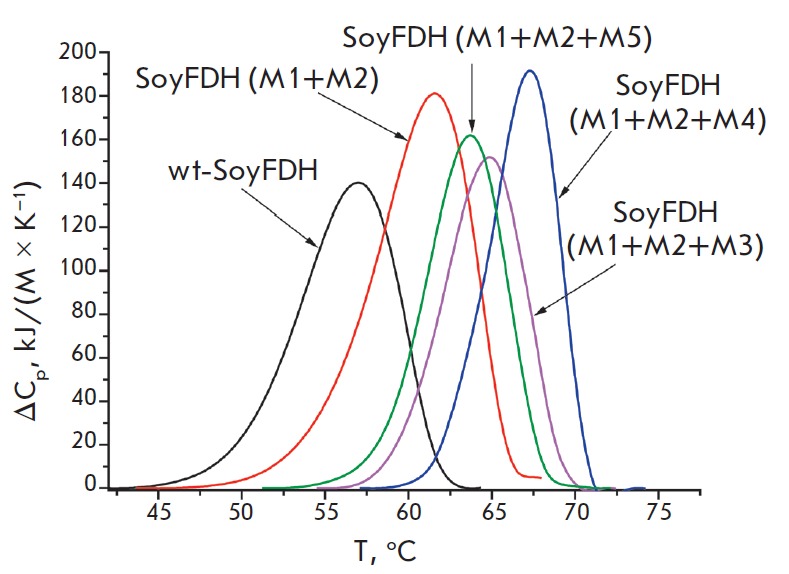
Differential scanning calorimetry melting curves for wild-type and multi-point mutant SoyFDHs. 0.1 M sodium phosphate buffer, pH 7.0. Enzyme concentration is 2 mg/mL, heating rate is 1 C/min. M1 – Ala267Met, M2 – Ile272Val, M3 – Phe290Asn, M4 – Phe290Asp, M5 – Phe290Ser

The results of the study of multi-point mutant Soy- FDHs by differential scanning calorimetry are presented in *Fig. 5*. For comparison, the melting curve of double-mutant SoyFDH Ala267Met/Ile272Val is also shown. *Fig. 5* demonstrates that an increase in the heat transition temperature in triple mutants compared to a double mutant has the same tendency as for determining the thermal stability through thermal inactivation kinetics: the higher the stabilization effect of a substitution at the 290^th^ position is, the higher the phase transition temperature of a triple mutant is. As expected, mutant SoyFDH Ala267Met/Ile272Val/ Phe290Asp proved to be the most stable one.

**Fig. 6:**
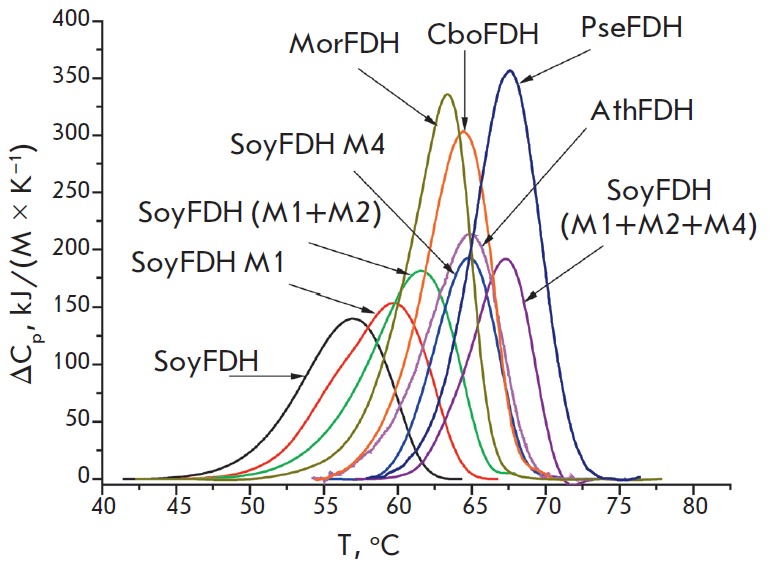
Differential scanning calorimetry melting curves for multi-point mutant SoyFDHs and wild-type formate dehydrogenases from various sources. 0.1 M sodium phosphate buffer, pH 7.0. Enzyme concentration is 2 mg/mL; heating rate is 1 C/min. M1–Ala267Met, M2 – Ile272Val, M3–Phe290Asn, M4–Phe290Asp, M5 – Phe290Ser. PseFDH, MorFDH, CboFDH, SoyFDH, and AthFDH are recombinant wild-type formate dehydrogenases from bacteria *Pseudomona*s sp.101 and *Moraxella *sp.C1, yeast *C. boidinii*, soybean *G. max*, and plant *A. thaliana*, respectively

*Fig. 6* shows melting curves for the most stable mutant SoyFDHs and enzymes from other sources that provide an assessment of the magnitude of a thermal stability increase in the mutants. It is evident that SoyFDH Ala267Met/Ile272Val/Phe290Asp is more stable than FDH from *A. thaliana*, *C. boidinii*, and* Moraxella *sp. C1 and is very close to the enzyme from* Pseudomonas *sp. 101 (PseFDH), which is one of the most stable described formate dehydrogenases [2, 15].

**Table 4:** Data of differential scanning calorimetry for wild-type and mutant formate dehydrogenases from various sources (0.1 M sodium phosphate buffer, pН 7.0)

Enzyme	Melting temperature, T_m_, °C	T_m_–T_m_^wt-SoyFDH^, °C	Cooperativity value, T_1/2_, °C	Reference
wt-PseFDH*	67.6	10.6	5.4	[17]
PseFDH GAV*	68.8	11.8	5.4	[17]
wt-MorFDH**	63.4	6.4	4.9	[17]
wt-AthFDH**	64.9	7.9	5.9	[17]
wt-CboFDH**	64.4	7.4	5.3	[17]
wt-SoyFDH**	57.0	0.0	7.1	[5]
SoyFDH М1 (A267M)	59.7	2.7	7.5	[4]
SoyFDHМ1+М2 A267M/I272V	61.6	4.6	6.8	[4]
SoyFDHF290D	64.8	7.8	5.0	[5]
SoyFDHF290N	61.3	4.3	6.6	[5]
SoyFDH F290S	59.9	2.9	6.4	[5]
SoyFDH М1+М2+М3 (A267M/I272V/F290N)	64.9	7.9	5.8	Present study
SoyFDH М1+М2+М4 (A267M/I272V/F290D)	67.3	10.3	4.8	Present study
SoyFDHМ1+М2+М5 (A267M/I272V/F290S)	63.7	6.7	5.6	Present study

* wt-PseFDH and PseFDH GAV are wild-type and mutant formate dehydrogenases from bacterium Pseudomonas sp.101,, respectively [2].

** wt-MorFDH, wt-CboFDH, and wt-AthFDH are wild-type recombinant formate dehydrogenases from bacterium Moraxella sp. C1, yeast C.boidinii, and plant A. thaliana, respectively.

*Table 4* shows the values of the thermal transition parameters. It is seen that SoyFDH Ala267Met/Ile272Val/ Phe290Asp has the highest phase transition temperature among all multi-point soybean FDH mutants, which agrees well with the data on the thermal inactivation kinetics. Comparison of this mutant form with formate dehydrogenases from other sources demonstrated that this enzyme ranks second after PseFDH for thermal stability.

Thus, we have produced three soybean mutant formate dehydrogenases that have a much higher thermal stability than the wild-type enzyme, as well as doubleand point-mutant precursors. The distinctive feature is that the effect is achieved without a significant change in the catalytic parameters compared to the original SoyFDH.

## References

[1] Tishkov V.I., Popov V.O. (2004). Biochemistry(Moscow)..

[2] Tishkov V.I., Popov V.O. (2006). Biomol. Eng..

[3] Alekseeva A.A., Savin S.S., Tishkov V.I. (2011). Acta Naturae..

[4] Alekseeva A.A., Savin S.S., Kleimenov S.Yu., Uporov I.V., Pometun E.V., Tishkov V.I. (2012). Biochemistry(Moscow).

[5] Alekseeva A.A., Serenko A.A., Kargov I.S., Kleimenov S.Y., Savin S.S., Tishkov V.I. (2012). Protein Eng. Des. Sel..

[6] Kargov I.S., Kleimenov S.Y., Savin S.S., Tishkov V.I., Alekseeva A.A. (2015). Protein Eng. Des. Sel..

[7] Romanova E.G., Alekseeva A.A., Pometun E.V., Tishkov V.I. (2010). Moscow Univ. Chem. Bull..

[8] Sadykhov I.O., E.G. I.O., Serov I.O., A.E. I.O., Yasnyi I.O., I.E. I.O., Voinova I.O., N.S I.O., Alekseeva I.O., A.A. I.O., Petrov I.O., A.S. I.O., Tishkov I.O., V.I. I.O. (2006). Moscow Univ. Chem. Bull..

[9] Andreadeli A., Flemetakis E., Axarli I., Dimou M., Udvardi M. K., Katinakis P., Labrou N.E. (2009). Biochim. Biophys. Acta..

[10] Slusarczyk H., Felber S., Kula M.R., Pohl M. (2000). Eur. J. Biochem..

[11] Felber S. (2001). Optimierung der NAD+-abhängigen Formiatdehydrogenase aus Candida boidinii für den Einsatz in der Biokatalyse.. Ph.D. Thesis. Heinrich-Heine University of Duesseldorf, 2001..

[12] Cornish-Bowden A. (2012). Fundamentals of Enzyme Kinetics. 4th Ed. Wiley-Blackwell, 2012. 510 p..

[13] Tishkov V.I., Uglanova S.V., Fedorchuk V.V., Savin S.S. (2010). Acta Naturae. 2010. V. 2. №. 2(5)..

[14] Serov A.E., Tishkov V.I. (2006). Moscow Univ. Chem. Bull..

[15] Rojkova A.M., Galkin A.G., Kulakova L.B., Serov A.E., Savitsky P.A., Fedorchuk V.V., Tishkov V.I. (1999). FEBS Lett..

[16] Serov A.E., Odintseva E.R., Uporov I.V., Tishkov V.I. (2005). Biochemistry (Moscow)..

[17] Sadykhov E.G., Serov A.E., Voynova N.S., Uglanova S.V., Petrov A.S., Alekseeva A.A., Kleimenov S.Yu., Popov V.O., Tishkov V.I. (2006). Appl. Biochem. Microbiol..

